# Epipericardial Fat Necrosis: A Concise Review of Literature

**DOI:** 10.7759/cureus.13106

**Published:** 2021-02-03

**Authors:** Meer R Zafar, Syed Farrukh Mustafa, Amir Shahbaz, Sami Warraich, Areeba Altaf

**Affiliations:** 1 Internal Medicine, Sisters of Charity Hospital, Buffalo, USA; 2 Internal Medicine, Jacobs School of Medicine and Biomedical Sciences, Buffalo, USA; 3 Internal Medicine, William Beaumont Hospital, Royal Oak, USA; 4 Internal Medicine, Sheikh Zayed Hospital, Lahore, PAK; 5 Internal Medicine, Icahn School of Medicine at Mount Sinai, Queens General Hospital, New York, USA; 6 Internal Medicine, Allama Iqbal Medical College, Lahore, PAK; 7 Internal Medicine, University at Buffalo, Buffalo, USA

**Keywords:** epipericardial, mediastinal, fat necrosis

## Abstract

Epipericardial fat necrosis (EFN) is an inflammatory process that occurs in the mediastinal fat surrounding the heart. It is a rare cause of acute chest pain and mimics more ominous clinical conditions such as acute coronary syndrome, aortic dissection, and pulmonary embolism. Clinicians are often not familiar with this condition due to its infrequent occurrence, and traditional textbooks of medicine and cardiology have not covered this topic adequately. In the past, EFN had been managed primarily with thoracotomy and surgical excision. This has changed with advances in imaging techniques and their more frequent utilization. Computed tomography (CT) of the chest is essential for the diagnosis of EFN as it allows for the evaluation of the nature and precise location of the lesion. Magnetic resonance imaging helps to differentiate EFN from other mediastinal fatty lesions such as lipomas or liposarcomas. The clinical presentation of acute chest pain along with CT findings of the encapsulated fatty pericardial lesion is adequate for diagnosis. Our review describes the emerging role of imaging in diagnosis and change in management over the last few years.

## Introduction and background

Fat necrosis in systemic adipose tissue can occur at various sites: in the breast and subcutaneous fat after trauma, peripancreatic fat in pancreatitis, and epiploic appendagitis [1,2]. Rarely, it may occur within the epipericardial fat. Epipericardial fat necrosis (EFN) is an inflammatory process that occurs within the epipericardial fat and leads to encapsulated fat necrosis [3]. It is an uncommon cause of acute chest pain and is a benign, self-limiting disease that mimics serious clinical conditions such as acute coronary syndrome and pulmonary embolism [4]. First reported in 1957, the condition is now better recognized due to advances in imaging techniques. Chest computed tomography (CT) typically shows a well-circumscribed ovoid fatty lesion with adjacent inflammatory changes in the cardiophrenic angle, more often on the left [5,6]. Conservative management leads to an improvement in symptoms. In this article, we will provide a concise review of the etiology, diagnosis, and management of EFN.

## Review

There has been some discrepancy in the literature regarding the nomenclature of EFN. Initially, Jackson reported it as pericardial fat necrosis in 1957, and multiple reports have used the same terminology in the following years [5]. However, Pineda et al. proposed that the necrosis occurs outside of the parietal pericardium and suggested that the term ‘epipericardial fat’ be used to classify these lesions [7]. Several other reports, including that of Bhatt et al. [8], have used the term mediastinal fat necrosis. In this article, we will use the term EFN. The exact prevalence of this condition is not well known due to its rare occurrence. Giassi et al. identified that among patients presenting to the emergency room with chest pain, 11 of 426 patients (2.15%) who underwent chest CT during their diagnostic workup had EFN [9]. The same study reported EFN in 0.26% of the patients undergoing chest CT for any cause. EFN seems to affect males more often than females with a wide age range, from 23 to 67 years [9]. There has been an increase in reporting of EFN in recent years, probably due to the utilization of imaging modalities, especially CT chest, over the last decade. On our comprehensive review, 61 cases have been reported in the English language literature.

Etiology

The pathophysiology of EFN is not well established, and two probable mechanisms may describe the underlying disease process. In some cases, the mass consisting of fatty tissue attached to the heart via a vascular pedicle, suggesting that acute torsion of it might cause the necrosis [5,10]. The alternative mechanism involves increased intrathoracic pressure and shearing forces during the cardiac cycle, particularly when the adipose tissue has underlying structural anomalies such as lipoma or hamartoma [11,12]. There are no established risk factors for EFN. Obesity was previously suggested as a risk factor, but subsequently reported cases showed no definite association [13].

Clinical presentation

EFN classically presents as chest pain in previously healthy individuals. The pain is pleuritic, and moderate to severe in intensity. The pain is predominantly left sided and some patients also experience dyspnea on exertion. Other systemic symptoms such as fever, myalgia, or cough have not been reported. The presentation is often similar to other common causes of chest pain, including myocardial infarction, pulmonary embolism, and pericarditis [4]. The pain gradually recedes and resolves over several days. Chester and colleagues reported syncope as an initial presentation in a patient with EFN [14]. Physical examination may reveal tenderness to palpation in the pericardium [10,15-17] and pericardial friction rub [10,14]. Laboratory tests such as creatine kinase and troponin are usually within normal limits [8]. Elevated C-reactive protein levels were reported in some cases, probably due to inflammation in the necrotic epipericardial fat [17-19]. Electrocardiogram, as well as echocardiography, is non-specific in most cases [3,8].

Image findings

**Figure 1:**
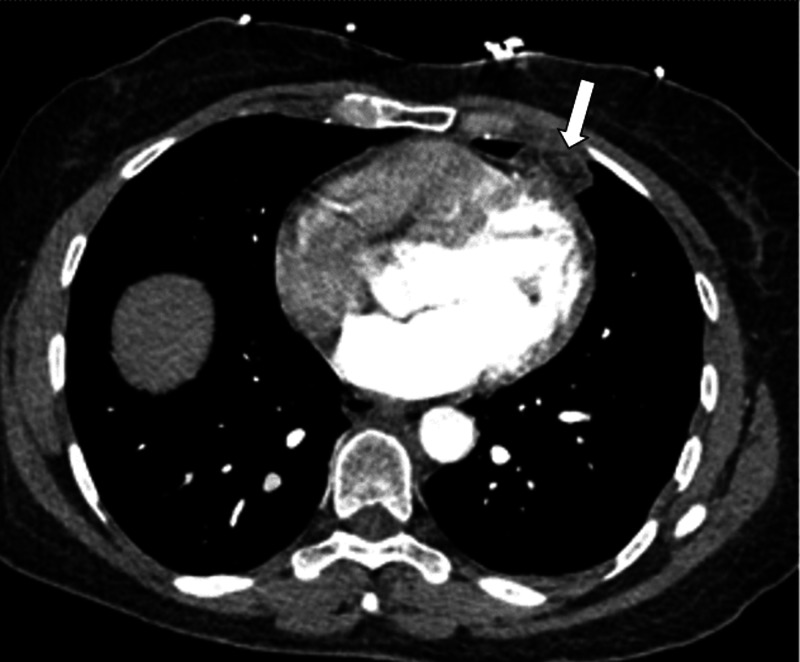
Computed tomography showing epicardial fat necrosis as ovoid-shape mass with a rim, central high attenuation focus, and surrounding soft tissue stranding as indicated by an arrow. Image provided by the Beaumont Hospital, Royal Oak with permission for use.

The workup usually begins with a chest x-ray. X-rays are suggestive but do not establish with certainty the diagnosis of EFN. The most common finding on X-rays is indistinct opacity, which usually develops along with the cardiomediastinal silhouette, raising concern for a mass lesion [7,19,20]. CT chest adds many more details and is vital to the diagnosis of EFN as it allows for the evaluation of the nature and precise location of the lesion. Ovoid-shape soft-tissue mass of adipose tissue density with a rim, central high attenuation focus, and surrounding soft tissue stranding is the typical CT finding of EFN (Figure 1) [21]. There is no invasion of the chest wall or myocardium. However, the lesion may extend into the pleural fissures as the epipericardial fat is continuous with them [5,8]. A similar pattern of stranding is also observed in fat necrosis in other tissues, including omental torsion and appendicitis [20]. Some of the lesions tend to develop peripheral or central calcifications [17]. The strandings along with a capsule surrounding the fatty tissue and the diaphragmatic integrity are strongly suggestive of EFN [19]. 

**Figure 2:**
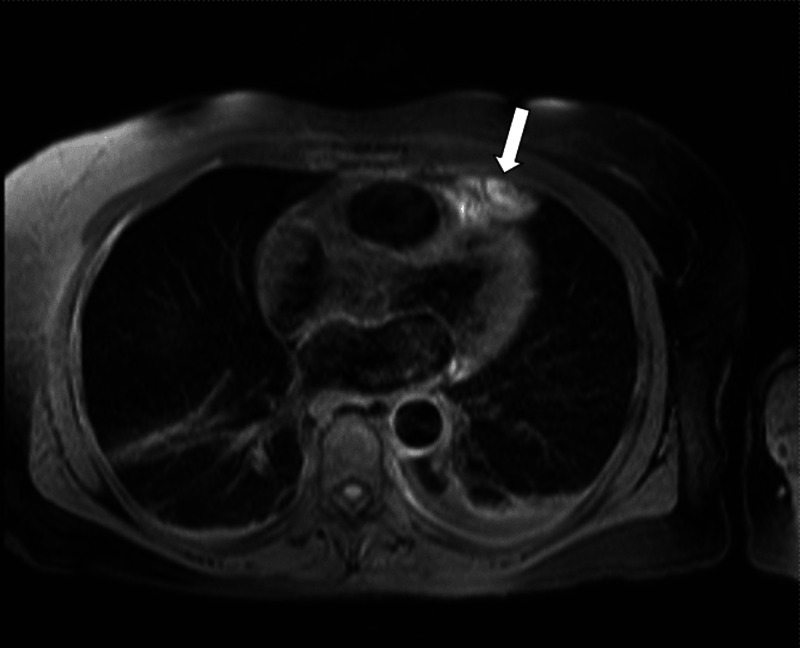
Magnetic resonance image T2-weighted high-intensity signal suggestive of an active lesion of epicardial fat necrosis as indicated by an arrow. Image provided by the Beaumont Hospital, Royal Oak with permission for use.

**Figure 3:**
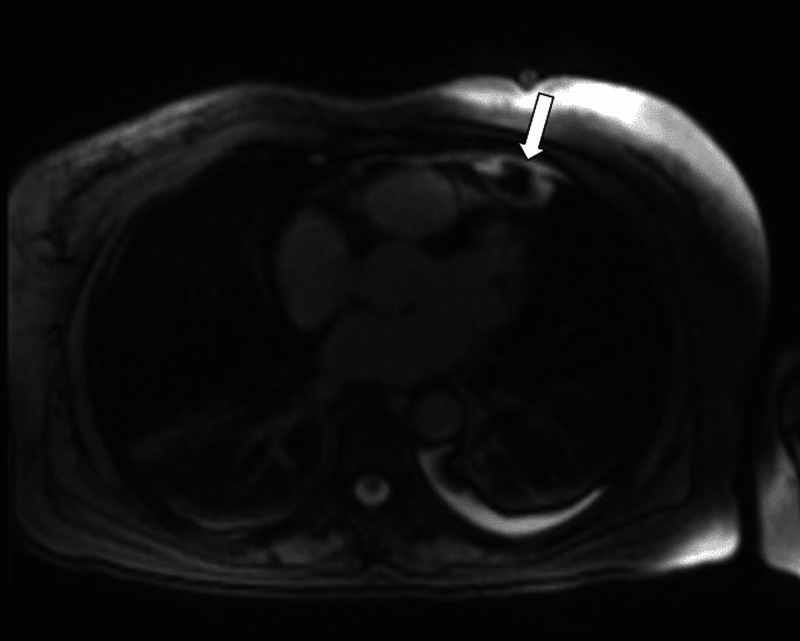
Magnetic resonance image T1-weighted high-intensity signal showing increased enhancement in the rim suggestive of fat necrosis as indicated by an arrow. Image provided by the Beaumont Hospital, Royal Oak with permission for use.

Differential diagnosis includes lesions such as thymoma, lipomas, liposarcoma, pericardial mesotheliomas, teratomas, and diaphragmatic hernias with abdominal fat occupying the cardiophrenic space [19,22]. Follow-up chest CT, weeks or months after the presentation, typically shows a marked decrease in the size of the mediastinal fat lesion and the adjacent pericardial thickening [23,24]. Magnetic resonance imaging (MRI) has been used less frequently in the diagnosis of acute EFN. Inoue et al. first reported a juxta-cardiac fat-density lesion on MRI, which he initially thought of as lipoma [25]. Baig et al. characterized EFN further as having high signal on T2-weighted MRI images suggestive of soft tissue edema and slight gadolinium enhancement characteristic of an active disease process (Figure 2) [13]. Lee et al. described a similar lesion with high intensity on T1- and T2-weighted MRI imaging that also had a low signal intensity in the peripheral rim and central dot-and-line area [23]. These findings are similar to those seen in patients with epiploic appendagitis [26]. Post-contrast T1-weighted images show increased enhancement in the rim, suggestive of fat necrosis (Figure 3) [27]. MRI helps to differentiate EFN from other mediastinal fatty lesions such as lipomas and liposarcomas. Echocardiographic findings of EFN had not well described in the literature until recently [8,24]. Diaz et al. reported sonograms of four cases, which showed an ovoid mass surrounded by a hypoechoic halo. The sonographic findings in epiploic appendagitis and lipo-necrosis of subcutaneous adipose tissue are similar to those seen in EFN [28,29]. EFN may display increased uptake on gallium Ga-67 scintigraphy. However, uptake is low-grade on 2-deoxy-2-[fluorine-18] fluoro-D-glucose positron emission tomography integrated with CT, findings that indicate the inflammatory process [30]. 

Histopathology

Exploratory thoracotomy has been performed frequently in the past for diagnosis of EFN and to rule out a neoplasm. Macroscopically, the lesion consists of circumscribed, lobulated adipose tissue fragments attached to the pericardium. The cut surface of the mass showed a white fibrous capsule enclosing degenerated fatty tissue [25,31]. Microscopic findings in EFN depend on the duration of lesion presence and stage of inflammation. Early lesions have hemorrhagic and liquefactive necrosis with central necrotic focus surrounded by neutrophilic infiltration and lipid-laden macrophages. Consequently, fibroblastic proliferation occurs, and the lesion surrounded by a collagenous scar [25]. At this stage, tissue contains lobulated areas consisting of necrotic fat cells, leaving only the ghost outlines of cells without nuclei. Granulation tissue with mild acute and chronic inflammatory infiltrate may also be recognized. The boundary consists of dense collagenous tissue with scattered infiltration of lymphocytes [25,31]. These findings are similar to those seen in fat necrosis in other body areas such as breast and epiploic appendages [31]. The necrotized fat may become detached and calcify and then present as a nodule that is sometimes mobile and shifts along with the pleural space [32].

Treatment

EFN had been managed primarily with thoracotomy and surgical excision in the past. This treatment led to the resolution of symptoms, with no significant postoperative complications or short-term mortality reported. A total of 20 cases of EFN, managed with thoracotomy and surgical excision, have been reported between 1957 and 2005. Inoue et al. suggested that surgery remains the first-line option to diagnose and manage EFN and rule out a thoracic neoplasm [25]. However, Pineda and colleagues, reported in 2005 a case of EFN, diagnosed non-invasively based on CT findings, and the patient was given symptomatic management with oral analgesics. The chest pain resolved within a few days, and complete resolution of the lesion was seen on a two-month follow-up CT [7]. Since then, there has been a gradual shift toward non-invasive, imaging-based diagnosis, and conservative approach toward management. Modern CT scan machines have improved spatial resolution allowing for better characterization of EFN characteristics. The clinical presentation of acute chest pain coupled with CT findings of the encapsulated fatty pericardial lesion with surrounding inflammatory reaction (pericardial thickening and dense strands) is sufficient for diagnosis by a qualified radiologist. However, due to its rare occurrence, the diagnosis may remain elusive in some cases. MRI can prove useful in such cases and can help differentiate EFN from other mediastinal lesions. Symptomatic treatment, usually with non-steroidal anti-inflammatory drugs for one to two weeks, leads to improvement in symptoms [13]. A follow-up CT scan is recommended in two to three months, and complete resolution of the lesion has been well documented [3,18,33-35]. There remains a role of thoracotomy and excision when the diagnosis remained uncertain and features suggestive of a thoracic neoplasm.

## Conclusions

EFN is an uncommon cause of acute chest pain and mimics other potentially life-threatening conditions presenting as chest pain. Our review highlights the recently emerging role of imaging in diagnosis and subsequent change in the management. The incidence of EFN will continue to rise due to the increasing quality and use of imaging techniques. Clinicians should be aware of this condition, which helps them to establish the diagnosis non-invasively and avoid unnecessary interventions.
